# In vitro cytotoxicity of human urine and its potential toxic parameters towards bladder cancer cells

**DOI:** 10.1371/journal.pone.0276127

**Published:** 2022-10-19

**Authors:** Hongda Zhao, Ryan Tsz-Hei Tse, Carol Ka-Lo Cheng, Christine Yim-Ping Wong, Angel Wing-Yan Kong, Ronald Cheong-Kin Chan, Peter Ka-Fung Chiu, Chi-Fai Ng, Jeremy Yuen-Chun Teoh

**Affiliations:** 1 Department of Surgery, S.H. Ho Urology Centre, The Chinese University of Hong Kong, Hong Kong, China; 2 Department of Anatomical and Cellular Pathology, The Chinese University of Hong Kong, Hong Kong, China; 3 European Association of Urology–Young Academic Urologists (EAU-YAU) Urothelial Carcinoma Working Group; PhD, PLOS, UNITED KINGDOM

## Abstract

**Background:**

Bladder cancer (CaB) has a high recurrence rate despite surgery. As bladder is constantly filled with urine, it is worthwhile to investigate whether it could have any detrimental effects on bladder cancer cells.

**Methods:**

We investigated the cytotoxicity of urine samples from CaB patients and normal controls on four CaB cell lines and tested the percentage of cell death, proliferation, adhesion, invasion and colonies formation ability. In order to identify the potential components involving in urine cytotoxicity, we evaluated some basic physiochemical parameters of urines, such as pH, osmolarity, creatinine (Cr), sodium (Na), potassium (K), chloride (Cl), calcium (Ca) and phosphate (PO_4_). We further compared the pH values of urine samples between CaB who developed recurrence versus those who did not. A more in-depth analysis on inflammatory markers was performed for two representative urine samples which demonstrated opposite cytoxic effects.

**Results:**

23 CaB patients and 20 normal controls were recruited into this study. According to *in vitro* experiments, both CaB and non-CaB urines had comparable effect on cell toxicity, proliferation, adhesion, invasion and colonies formation ability in four cell lines, HTB9, RT4, T24 and UMUC3, while RT4 was the most sensitive to urine toxicity. After evaluating the relationship between basic physiochemical parameters and cytotoxicity, we found out that there were strong negative correlations between pH value and 24 hours death rate for the 4 CaB cell lines (HTB9 r = -0.6651, p<0.001; RT4 r = -0.8335, p<0.001; T24 r = -0.4924, p<0.001; UMUC3 r = -0.7066, p<0.001). Osmolarity, urine Cr and PO_4_ all had weakly or moderately positive correlations with CaB cells on 24 hours death rate. CaB patients who developed recurrence had more alkaline urine than those who did not develop recurrence. In the urine sample with the highest cytoxicity, high concentrations of IL-6 and IFN-gamma were found.

**Conclusions:**

Our study confirmed that there was not statistically significant difference in cytotoxicity between CaB and non-CaB urines. However, we identified some parameters that could have an impact on cytotoxicity towards CaB cells. Modifying certain urine characteristics peri-operatively may induce cytotoxicity, avoid tumour re-implantation, and reduce the chance of cancer recurrence.

## 1. Introduction

Urinary bladder cancer is the 9th most common cancer worldwide with approximately 550,000 new cases diagnosed and 220,000 deaths every year [1]. Despite the progress in diagnostic techniques and the improvement in surgical and nonsurgical therapies, non-muscle-invasive bladder cancer (CaB) has a high recurrence rate [2, 3]. As a result, bladder cancer imposes a significant burden to the healthcare system.

Transurethral resection of bladder tumor is the current standard in managing non-muscle-invasive CaB [4]. Although transurethral resection is safe and relatively simple to perform, many carcinoma cells can be released into the urine during the resection procedure [5–7]. The floating tumour cells may re-implant to the bladder wall resulting in early disease recurrence. As bladder is an organ for urine storage, these floating tumour cells are constantly in touch with the urine inside the bladder. It is therefore worthwhile to investigate whether certain urine characteristics could have detrimental effects on bladder cancer cells, hence reducing its chance of tumour re-implantation and cancer recurrence.

As early as 1989, the protective role of urinary purified TNF binding protein was reported in murine A9 cells and human foreskin fibroblasts, FS11 [8]. Arsenic metabolites are common component in human urine, and its cytotoxicity was confirmed in human bladder cancer cell line EJ-1 [9]. More recently, urine from patients with interstitial cystitis has been reported to be more cytotoxic than urine from healthy subjects when tested in vitro against HTB-4 urothelial cells. Overall, whether human urine impose any cytotoxic effect to bladder cancer cells is controversial; the potential role of urine in *in vitro* tumorigenicity is worthwhile to explore [10].

In this study, we hypothesized that certain urine characteristics could have detrimental effects on CaB cells and bladder cancer patients’ urine would be more favorable for bladder cancer cells to grow. We first investigated the cytotoxicity of human urine from bladder cancer (CaB) patients and non-CaB patients on four bladder cancer cell lines. In terms of *in vitro* tumorigenicity, percentage of cell death, proliferation, adhesion, invasion and colonies formation ability were investigated. Basic physiochemical parameters of urines, such as pH, osmolality, sodium Na, potassium K, calcium Ca, chloride Cl, phosphate PO_4_ and creatinine Cr were evaluated to understand the correlation of physiochemical properties to urine cytotoxicity. Furthermore, several inflammatory factors, such as Interferon-gamma (IFN-gamma), Interleukin-6 (IL-6), Interleukin-8 (IL-8), Nerve growth factor (NGF) and Tumour Necrosis Factor alpha (TNF-alpha) were also tested via ELISA. We believe these results may provide valuable insights on how we can manipulate the fluid environment in order to optimise the oncological outcomes.

## 2. Patients and methods

### 2.1. Cell lines and reagents

Four human urinary CaB cell lines (T24, UMUC3, RT4 and HTB9) were used in this study. T24 and RT4 cell lines were maintained in McCoy’s 5A Medium with high-glucose and L-glutamine (ThermoFisher Scientific Inc.). UMUC3 and HTB9 were grown in Minimum Essential Medium (MEM) (ThermoFisher Scientific Inc.) and in Roswell Park Memorial Institute (RPMI) 1640 Medium with L-glutamine (ThermoFisher Scientific Inc.) respectively. All the cells were stocked in liquid nitrogen in dimethyl sulfoxide (DMSO) for permanent storage until use. The cell experiments were performed within 40 passages of the cells. After trypsinization, 1x10^6^ T24, UMUC3, RT4 and HTB9 cells were prepared in suspension for patient urine analysis. Cells were then centrifuged and cell viability was assessed by 0.4% Trypan Blue dye exclusion test. Living cells were further tested on proliferation assay, adhesion assay, invasion assay and colonies formation assay; and all assays were performed in triplicate wells.

### 2.2. Patients and follow-up regimen

The study was approved by the Ethics Committee of the Chinese University of Hong Kong. CaB patients with superficial bladder cancer and normal controls were recruited after informed written consent had been obtained. All patients underwent flexible cystoscopy to define its bladder cancer status. And we classified the bladder cancer into high- and low-grade disease based on standardized histomorphologic features as described by the World Health Organization. Follow-up time was calculated from the date of diagnosis to the first of either recurrence. If patients kept alive without any disease recurrence, they were censored at the date of their last follow-up.

### 2.3. Specimens

We selected 23 CaB and 20 non-CaB urine samples from our biobank, and all urine samples were freshly collected and filtered sterile for cells incubation. Urine was incubated with cells at 37°C for 1 hour, and then prepared for downstream proliferation assay, adhesion assay, invasion assay and colony formation assay. Continuous incubation of urine with cells for 24 hours was assessed for cell viability. Urine parameters such as pH value, Osmolarity, urine creatinine (Cr), sodium (Na), potassium (K), chloride (Cl), calcium (Ca) and phosphate (PO_4_) were measured and recorded. We further compared the pH values of urine samples between CaB patients who developed recurrence versus those who did not.

### 2.4. Proliferation assay

The growth rates of T24, UMUC3, RT4 and HTB9 cells were tested by the proliferation assay. Living cells were first seeded in 96-well plates (100 μl/well) at a density of 1x10^4^ cells/mL. After seeding and incubation for 24 and 48 hours, cells were treated with 10 μl 3-[4,5-dimethylthiazol-2-yl]-2,5-diphenyltetrazolium bromide (MTT) (ThermoFisher Scientific Inc.) and incubated for 4 hours. MTT-mixture was removed and DMSO was added to dissolve the purple formazan produced by reduction of MTT by living cells and the absorbance was measured at 590 nm using a microplate reader.

### 2.5. Adhesion assay

Adhesion assay was performed by plating 2x10^4^ cells/mL on Gibco® Collagen I-coated plate (ThermoFisher Scientific Inc.). Cells were centrifuged at 71g for 5 minutes at room temperature, followed by incubation for 15 minutes at 37°C in a humidified incubator with 5% CO_2_. After incubation, media in each well containing cells was then removed and the whole plate underwent centrifugation upside down at 7g for 5 minutes at room temperature. A MTT assay was then performed. After incubation for 4 hours, MTT-mixture was removed and DMSO was added, the absorbance at 590 nm was measured by a microplate reader.

### 2.6. Invasion assay

Cell invasion assays were carried out using BD BioCoat Matrigel Invasion chambers (BD Biosciences, USA) according to manufacturer’s protocol. Briefly, 5x10^4^ cells/mL living cells were seeded in each transwell containing 0.5% serum medium. The transwell insert was plated onto the 24-well plated containing 5% serum medium as a lower compartment, serving as an attractant for the cells to invade from the upper compartment towards to lower compartment through the microporous Matrigel® matrix-reconstituted membrane on the basement of each transwell insert. After incubation at 37°C in a humidified incubator with 5% CO_2_ for 48 hours, the transwell insert was removed and crystal violet stain was performed, accumulating the cell nuclei of the invasive cells which could enzymatically degrade the Matrigel® matrix and invade through membrane pores to the lower compartment. Stained cells were photographed by an inverted microscope and counted by the Image J software in three randomly selected fields.

### 2.7. Colony formation assay

To assess the cell’s ability to grow into a colony, 1000 living cells were seeded onto a 100 mm plate in their respective culture media. The plates were then incubated at 37°C in a humidified incubator with 5% CO_2_ for 9 days. After the incubation, crystal violet stain was performed, and the living cells were indicated by the total number of stained cell clones on the 100 mm plates. At least 50 cells were required to define a colony.

### 2.8. Inflammation biomarkers

IL-6, IL-8, NGF, TNF-alpha and TNF-gamma were analyzed by enzyme-linked immunosorbent assay (ELISA) following the manufacturer’s instructions (Cloud-Clone Corp, USA) and results were expressed as pg/mL.

### 2.9. Statistical analysis

Data are plotted as mean ± standard error of the mean (SEM). Statistical analysis was performed by Student t-test for independent samples with equal variances. The level of significance was set at p<0.05 as *, and p<0.01 as **. Pearson’s correlation coefficient (r) was used to evaluate degree of correlation between cytotoxicity and chemical parameters. The coefficient of determination (R2) was utilized to assess the ability of the correlation to account for variance in data. A P-value of <0.05 was considered significant. All statistics were completed utilizing Microsoft Excel (Microsoft, Redmond, WA) or GraphPad PRISM (GraphPad Software Inc, La Jolla, CA).

## 3. Results

### 3.1. Human urines impose cytotoxicity in CaB cells

**Fig 1:**
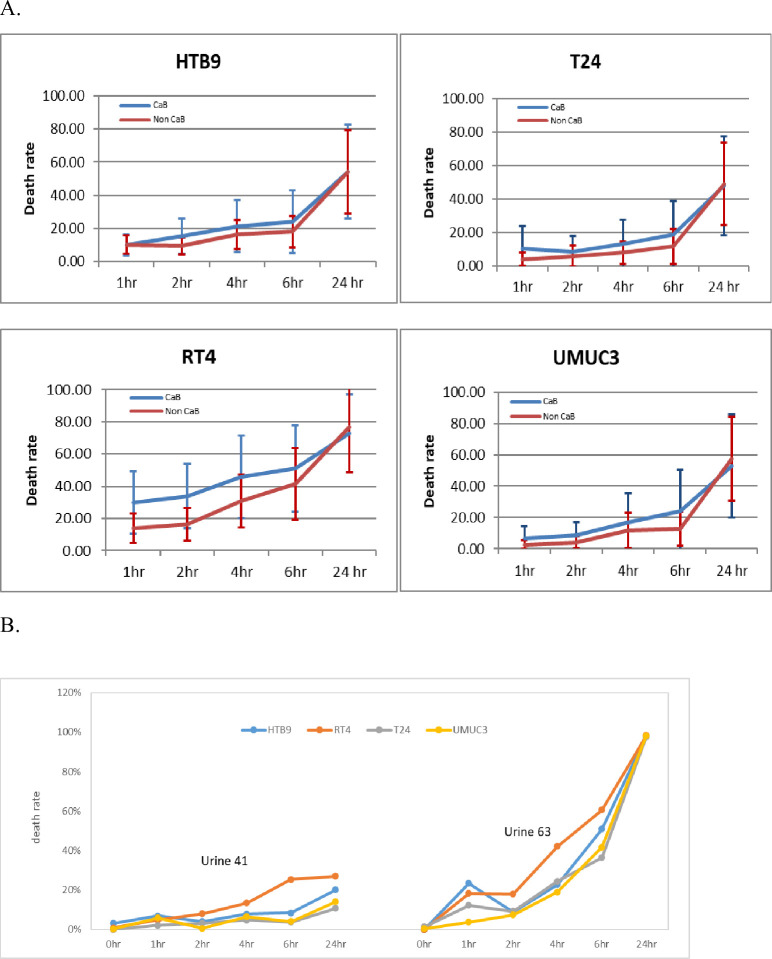
Urine toxicity on CaB cell lines by viability assay. (A) Four CaB cell lines HTB9, RT4, T24 and UMUC3 were tested. 23 CaB urine and 20 non-CaB urine were collected for the viability assay. 1x10(6) cells/ml suspensions were incubating with 0.22um filter sterile urine, and cells were counted at 1, 2, 4, 6 and 24 hours with Trypan blue stain. Data was presented as percentage of dead cells. (B) Two patient urines showed significant result, CaB urine U41 was the least toxic and non-CaB urine U63 was the most toxic to all four cell lines. All cells count were done in triplicate.

To understand the *in vitro* toxicity of human urines, we collected 23 urine from CaB patients and 20 urine from non-CaB patients. 0.22 μm filtrated sterile urine was added to suspension cells and result showed that CaB urines had similar toxicity on all four CaB cell lines, compared with non-CaB urines (Fig 1A). For *in vitro* cytotoxicity IC50, HTB9, T24 and UMUC3 were well tolerant to both CaB and non-CaB urines, with IC50 value of around 24 hours incubation. RT4 was more sensitive to urine toxicity with IC50 at 6 hours for CaB urines and around 10 hours for non-CaB urines (Fig 1A). We also compared cell death rate between CaB and non-CaB urines at different time point, and found out that CaB urine’s toxicity is in general higher than non-CaB. For RT4, CaB urine appeared to be more cytotoxic to RT4 than non-CaB urine, particular from 1 hour to 6 hours incubation (1 hour p<0.01, 2 hours p<0.01, 4 hours p<0.05). However, at 24 hours, the cell death rates between CaB and non-CaB groups were largely comparable. On the other hand, among 43 urines samples being tested, CaB U41 is the least toxic one to all 4 CaB cell lines at death rate below 30% after 24 hours incubation, while non-CaB U63 is the most toxic urine at death rate 74% to T24 and death rate larger than 90% to HTB9, RT4 and UMUC3 (Fig 1B). Our results showed that different urine samples could impose various degrees of cytotoxicity towards different CaB cell lines.

### 3.2. Human urines regulate cancer property in CaB cells

**Fig 2:**
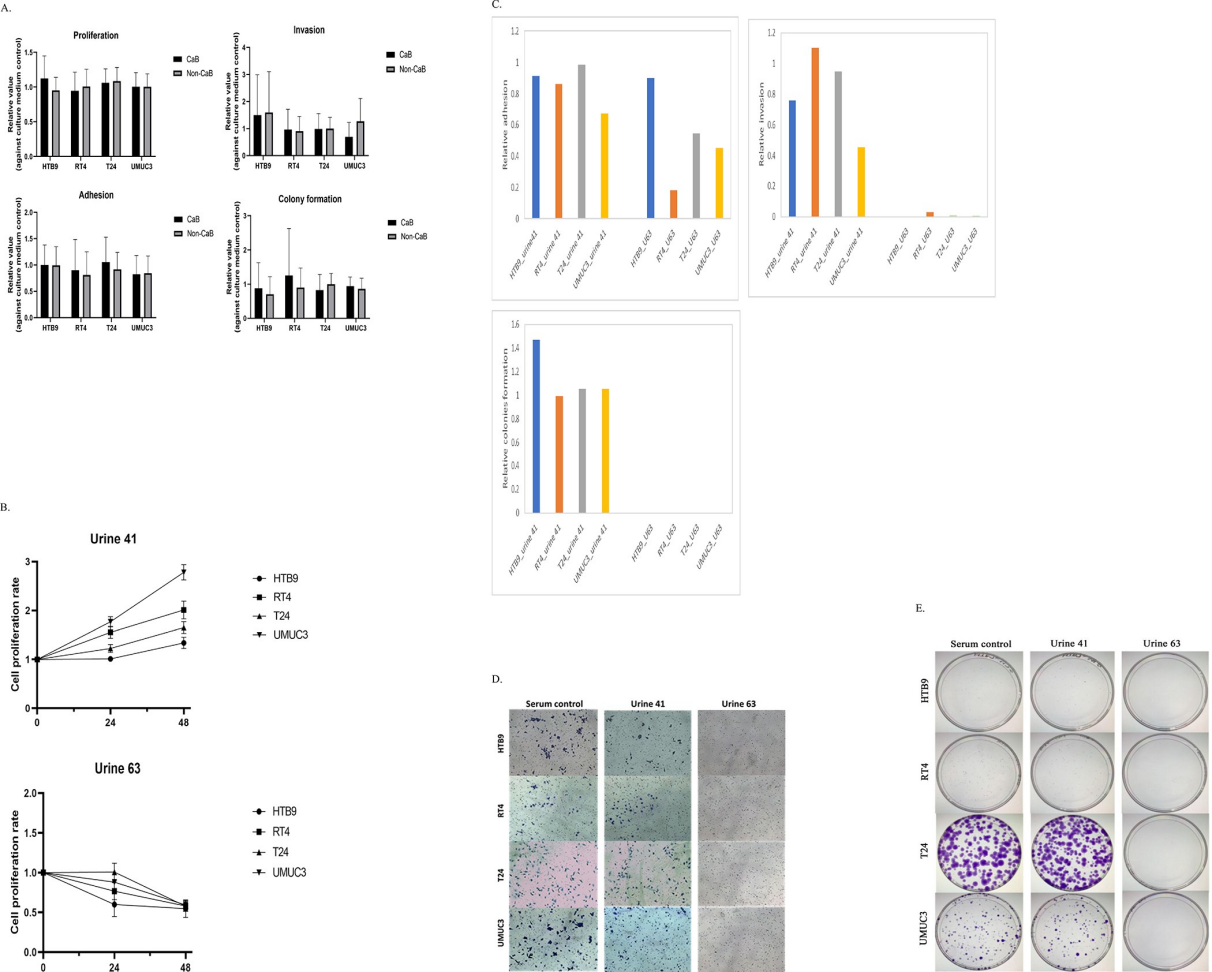
Tumorgenicity properties (A) After incubating in urine for 1 hour, 1x10(6) living cells were collected for proliferation, adhesion, invasion and colonies formation assay. CaB and non-CaB urines showed similar toxicity in all four cell lines. Data was presented as relative value against culture medium control. (B) CaB U41 and non-CaB U63 were showing significant result on all four cell lines. For proliferation assay, living cells were evaluated by MTT. U41 promoted cell proliferation and U63 suppressed or killed all cells for all four cell lines. (C) For adhesion assay, adhesion ability was evaluated by measuring the attached cells by MTT assays and OD reading was proportional to the number of adhered cells. U41 preserved the adhesion ability of HTB9, RT4 and T24, while U63 inhibited the cell adhesion of RT4 significantly. (D) To evaluate the invasion ability, cells passing through the matrigel were stained with Crystal Violet and total number of invasive cells were counted using the ImageJ software. (E) Colonies formation abilities were determined by seeding 1000 living cells onto a 100mm plate and incubated for 9 days. Colonies were then stained with crystal violet and colonies with more than 50 cells were counted. All data above was presented as relative value against culture medium control and all assays were done in triplicate.

To examine the impact of human urines on in vitro tumorigenic properties, after 1 hour incubation in urines, suspension cells were collected for proliferation, adhesion, invasion and colonies formation assays. Our results showed that both CaB and non-CaB urines had comparable effects on the four assays in all four cell lines (Fig 2A). Among 43 urine samples tested, CaB U41 promoted cell proliferation but non-CaB U63 suppressed cell growth in all four CaB cell lines (Fig 2B). U41 treatment preserved most adhesion ability of HTB9, RT4 and T24, while U63 incubation suppressed cell adhesion significantly in RT4 (Fig 2C). U41 treatment preserved the invasion ability of HTB9, RT4 and T24; and preserved the colonies formation ability in all four cell lines. Interestingly, U63 had the strongest toxicity to all CaB cell lines and inhibited invasion and colonies formation of all HTB9, RT4, T24 and UMUC3 (Fig 2D and 2E). Our results suggested that human urine could regulate cancer cells properties and change the in vitro tumorigenic properties.

### 3.3. Chemical parameters of human urines contribute to urine toxicity

**Fig 3:**
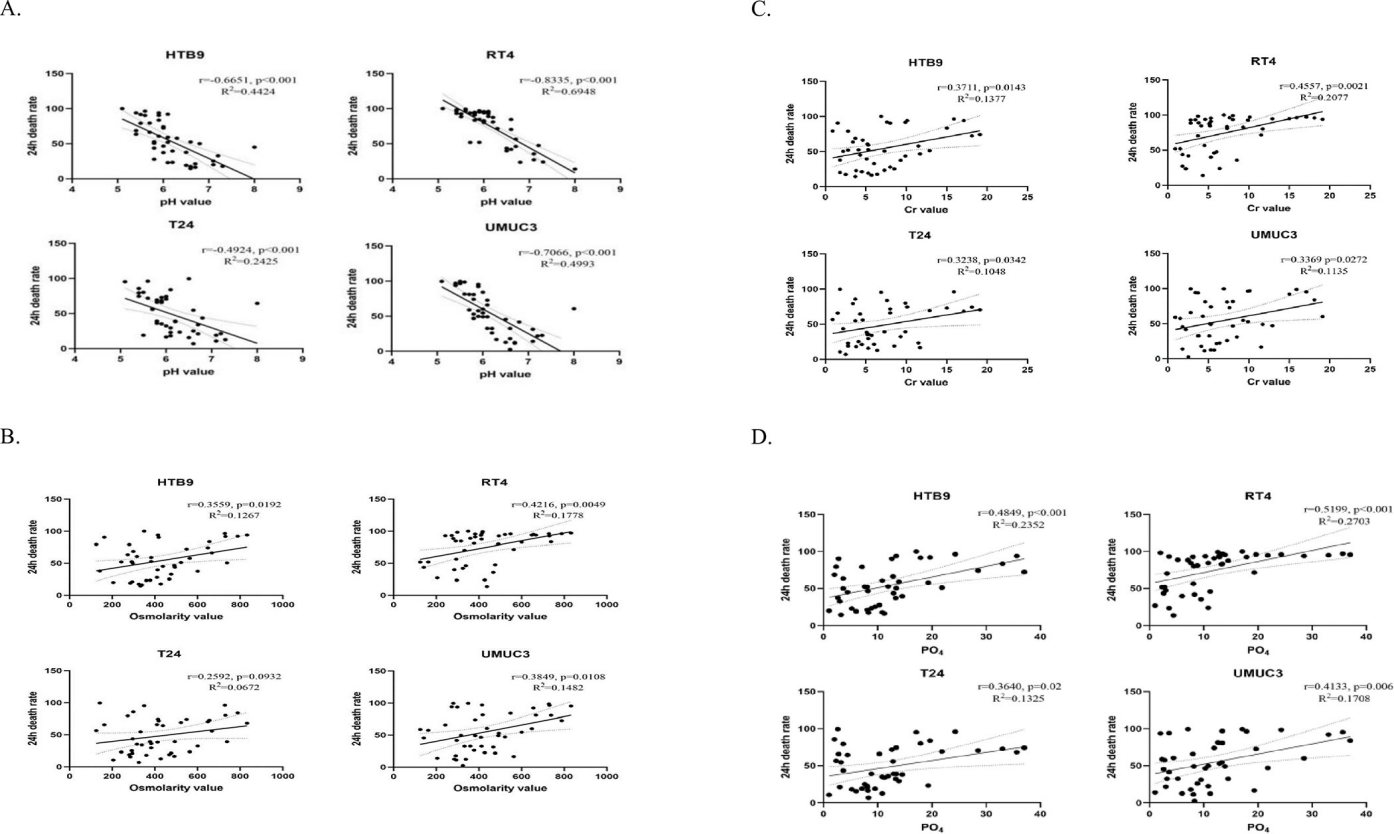
Correlation between the cancer cells’ 24h death rate and different chemical parameters of urine. (A) pH value strongly correlated with 4 cancer cell lines’ death. Urine’s (B) osmolarity, (C) Creatinine Cr and (D) phosphate PO_4_ value had a moderate and positive relationship with bladder cancer cells’ death, thus increasing human urine’s cytotoxicity.

**Fig 4:**
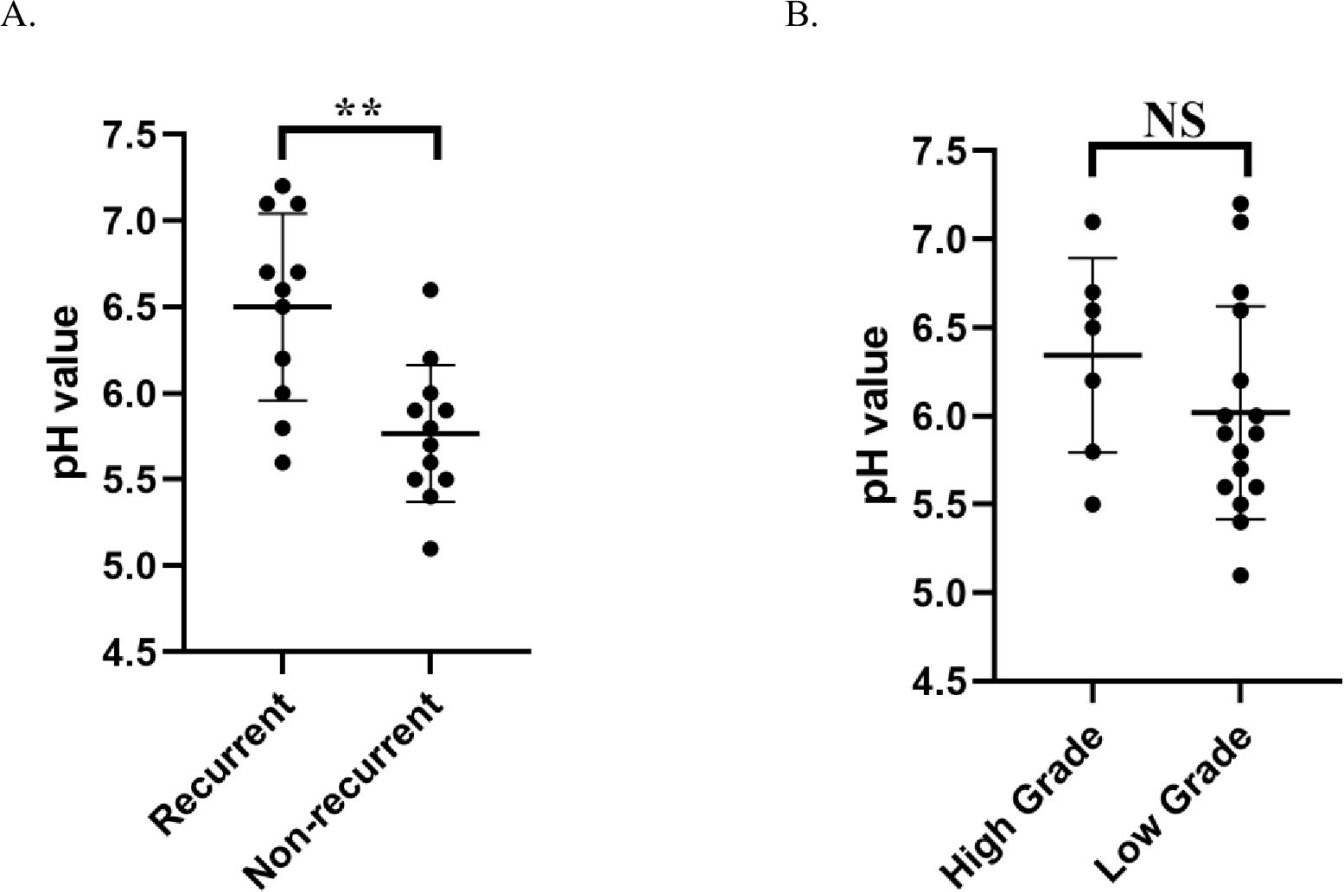
The pH value of urine between (A) recurrent and non-recurrent group, and (B) high-grade and low-grade tumor group.

We found out that urine pH, osmolarity, PO_4_and Cr value had signficant correlations with cytotoxicity. For pH value, there were strongly negative correlations with 4 CaB cell lines’ 24h death rate(HTB9 r = -0.6651, p<0.001; RT4 r = -0.8335, p<0.001; T24 r = -0.4924, p<0.001; UMUC3 r = -0.7066, p<0.001) (Fig 3A), which means that acidic urine could induce cancer cell death. Osmolarity value (Fig 3B), urine Cr (Fig 3C) and PO_4_ (Fig 3D) all had positive correlations with CaB cells’ 24h death rate. The results were consistent across all four CaB cell lines. We further compared the pH values of urine samples between CaB patients who developed recurrence versus those who did not. We found out that recurrent group had more alkaline urine than non-recurrent group (p = 0.001) (Fig 4A), but there was no significant difference between high-grade and low-grade bladder cancer (Fig 4B). In summary, our results proved that certain chemical parameters could contribute to urine toxicity, thus influencing cancer cells properties and oncological outcome.

### 3.4. Inflammatory markers, chemical parameters and clinical information for U41 and U63

As our results showed that CaB U41 promoted cell proliferation and preserved most adhesion ability of bladder cancer cell lines, but non-CaB U63 suppressed cell growth and adhesion. In order to investigate the underlying mechanisms, we tested the concentration of IFN-gamma, IL-6, IL-8, NGF, TNF-alpha in urine (Table 1) and found out that U63 had higher concentration of IL-6 and IFN-gamma than U41, and lower concentration of IL-8. The levels of NGF and TNF-alpha were comparable between the two urine samples. In addition, non-CaB U63 had lower pH value and higher osmolarity (Table 1). Together with extreme pH and high osmolarity, higher amount of IL-6 and IFN-gamma were suggested to contribute to the cytotoxicity of U63.

**Table 1:** Inflammatory markers and chemical parameters for U41 and U63.

Sample ID	IFN-gamma (pg/ml)	IL-6 (pg/ml)	IL-8 (pg/ml)	NGF (pg/ml)	*TNF-alpha (pg/ml)*	pH	Osmolarity (mmol/L)
U41	<1	0.164	56.41	<1	3.34	7.1	204
U63	10.62	7.8	6.013	<1	2.54	5.4	414

## 4. Discussion

In this study, we aimed to investigate the effects of urine on CaB cell lines. Four CaB cell lines in three subtypes, namely RT4 (luminal), HTB9/5637 (basal), T24 and UMUC3 (non-type), were used to explore their unique cellular reaction in different culture reagents. 23 CaB and 20 non-CaB urine samples were collected for the experiments. We found that there was no significant difference in cellular toxicity between CaB and non-CaB urines. While cytotoxicity could differ across different urine samples, its cytotoxic effects on the different CaB cell lines are usually consistent within the same urine sample. Among the four CaB cell lines, RT4 was most sensitive to urine toxins. Regarding the IC50 of the four CaB cell lines bathing in urine, only RT4 showed IC50 at around 7 hours, while HTB9, T24 and UMUC3 reached 50% death rate at around 24 hours. RT4 belongs to luminal subtype in molecular characterization and owns a relatively weak oncogenic property [11], and this might be explained by their sensitivity towards urine toxicity [12, 13]. On the other hand, we found that basal subtype and non-type bladder cancer cell lines exhibited higher tolerance to urine cytotoxicity. Regarding the tumorigenicity properties such as proliferation, adhesion, invasion and colonies formation, bathing the cell lines in most urine samples for one hour did not result in any significant adverse effects. In conclusion, most patient urine did not induce strong cytotoxicity towards the CaB cancer cell lines.

Among the 43 urine samples tested, there were two representative samples, U41 and U63, which showed dramatically different results. CaB U41 was collected from a male patient with bladder cancer and received TURBT without any post-operative intravesical therapy. Upon our collection of his urine in 2018, the patient did not suffer from any major diseases and he had not taken any drugs or medications. On the other hand, non-CaB U63 was collected from a male patient with no history of CaB. He was an ex-smoker and had past history of hypertension, diabetes mellitus, low urinary tract symptoms, prostate hyperplasia, elevated PSA and anaemia. Two months before his urine collection, he was given antibiotics, amoxicillin and metronidazole, to treat infections in urinary tract. The medicine he received was also commonly given to the other 42 patients. CaB U41 was the most non-toxic urine towards cancer cells with death rate at 10–27% after 24 hours incubation. Bathing cancer cells in U41 not only had no effects in the tumorigenic properties, but actually promoted proliferation in all four cell lines. On the other hand, non-CaB U63 was the most toxic urine sample. After 24 hours of incubation, U63 killed 75% of T24 and over 90% of HTB9, RT4 and UMUC3. For tumorigenicity assays, one hour bathing of cancer cells in U63 totally suppressed invasion and colonies formation in all four cell lines. We believe these results might be explained by the differences in the urine characteristics, which were further investigated in the latter part of our experiments.

Urine consists of water, urea (from amino acid metabolism), inorganic salts, creatinine, ammonia, inflammatory factors and pigmented products of blood breakdown, one of which (urochrome) gives urine its typically yellowish colour. In order to understand human urine properties and its correlation with cytotoxicity, eight basic physiochemical parameters including pH, osmolarity, sodium (Na), potassium (K), calcium (Ca), chloride (Cl), phosphate (PO_4_), creatinine (Cr), were tested. We found that pH, osmolarity, PO_4_ and creatinine Cr were involved in direct cell toxicity, in terms of cell death rate 24 hours after urine incubation. The role of pH toxicity was the most significant with p-value <0.001 on all four cell lines, and CaB patients who developed recurrence had more alkaline urine than those who did not develop recurrence. Normal urine is reported to be slightly acidic with usual values between 6.0–7.5 [14]. Alteration in pH values may possess adverse biological consequences to cancer cells. von Euler *et al*. investigated cell lines responses in different pH levels. The authors observed that cell lines under acidic environment, pH 3.5–5.0, showed abnormal and apoptotic morphology, such as shrunk, condensed chromatin, nuclear fragmentation, lost cell-to-cell contact and in part had detached from the plate. Of note, intracellular acidification may upregulate the activity of DNAse II, optimal pH of 5.0, resulting DNA digestion and fragmentation in apoptosis. On the other hand, necrosis was also induced under high pH [15]. As we all know, some drugs and diet can affect the pH value of urine. For example, Diuretics and high protein foods can decrease urine pH (more acidic), whereas diets high in fruits and vegetables can increase urine pH (more alkaline). Therefore, by adjusting fluid environment during and after urological surgeries, proliferation of cancer cells can be controlled as to prevent re-implantation and reduce recurrence rate.

In addition to pH levels, in terms of death rate, phosphates also induced cell toxicity. Our bodies require phosphates for bones and teeth building and repairment. Nonetheless, excess amount of phosphates will be filtered out and excreted through urine. Therefore, we may can adjust urine phosphates via diet, such as eating more high protein foods. Macro *et al*. reported that hyperphosphatemia induced endothelial cell lines shrinkage, membrane blebbing and lost attachment to substratum, which are evidence of apoptotic events. High phosphate concentrations also increased ROS generation and caspase activation although underlying mechanisms remained elusive [16]. As a fact that intracellular phosphates are regulated by mitochondria, excess amount of phosphates may cause disruption to mitochondrial membrane potential, therefore, initiating apoptosis. These suggested phosphates concentration was an essential death signal to cells. The above study was in line with our experimental results that phosphate concentrations may alter cell proliferation rate. These days, regulating extracellular osmolarity is becoming a promising method for cancer treatment, as several studies demonstrating its cytocidal effects on cancer cells [17]. The in vitro and in vivo studies indicated the cytocidal effects of changing liquid osmolarity on esophageal, gastric, colonic, pancreatic, and liver cancer cells. In addition, common cytokines such as IFN-gamma and IL-6 may contribute to urine cytotoxicity, and could play an important role in tumor micro-environment [18]. For example, as a cytokine which takes part in promoting innate and adaptive immune responses, IFN-gamma also has the ability to prevent the development of primary carcinogen-induced sarcomas [19]. For IL-8, it has many pro-tumor functions, such as angiogenesis, cancer stem cell survival signaling, and the recruitment of myeloid cells with the potential to immunosuppress and supply growth factors locally [20].

## 5. Limitations

Our study investigated the cytotoxicity of human urine and the underlying chemical properties of urines that could regulate cellular tumorigenicity. In order to investigate urine property and its induced toxicity, 8 common physiochemical parameters were tested. However, chemical composition of urine is complicated; it includes variety of metabolites, ions, organic solutes, glucose and protein. Urine component is strongly affected by dietary and environmental factors. It is difficult to understand the patients’ habit comprehensively and we may overlook some important factors that contributes to urine toxicity. Although we found that pH has a significant effects in 24 hours-cell dead rate, pH will not be the only factor that induces cell death, therefore a more comprehensive analysis of urine components is necessary. Besides, our study only focused on the effects being tested on CaB cell lines. Cancer cell lines cannot fully mimic the complicated tumor microenvironment *in vivo*, especially the interactions among different cell types. To further investigate how different physiochemical parameters correlates and interacts with the *in vivo* TME, future experiments on 3D models which can replicate TME, such as organoid cultures are required. Moreover, our study investigated only a small number of patients urine samples; a larger cohort validation becomes particularly important in future studies. Last but not least, levels of different parameters maybe altered by various factors, such as stages, grades, size of tumors and other complications. Further efforts are required to standardize and adjust levels of physiochemical parameters based on these factors.

## 6. Conclusions

Bladder cancer is a common cancer worldwide. Currently, TURBT is the golden standard to resect NMIBC tumor, however, it is accompanied with high cancer recurrence rate. Our findings confirmed that there was no difference in cytotoxicity between CaB and non-CaB urines on the 4 CaB cell lines; HTB9, RT4, T24 and UMUC3. Different urine could have very different cytotoxicity, and we identified four parameters, namely urine pH value, osmolarity, phosphate PO_4_ and creatinine Cr that may have an impact on cytotoxicity. It is possible to modify the specific urine factors per-operatively to induce cytotoxicity and avoid cancer cells re-implantation, and therefore reduce the chance of cancer recurrence in the future.

## Supporting information

S1 Table Clinical information for bladder cancer patients. (DOCX)
