# Supplementary material for: In vitro cytotoxicity of human urine and its potential toxic parameters towards bladder cancer cells
Source: PLoS One. 2022 Oct 19;17(10):e0276127. doi: 10.1371/journal.pone.0276127 (PMC9581372; doi:10.1371/journal.pone.0276127)
Supplement: S1 Table — (DOCX) [file pone.0276127.s001.docx]

S1 Table. Clinical information for bladder cancer patients

|  | **ID** | **Age** | **Gender** | **BMI** | **TURBT** | **Tumor Stage** | **Tumor Grade** | **Recurrence** |
| --- | --- | --- | --- | --- | --- | --- | --- | --- |
| **CaB** | U18 | 66 | Female | 29.80 | Yes | Ta | LG | No |
|  | U19 | 64 | Male |  | Yes | T1 | LG | No |
|  | U20 | 63 | Female |  | Yes | Ta | LG | No |
|  | U27 | 85 | Male | 21.08 | Yes | Ta | LG | No |
|  | U31 | 38 | Female | 28.10 | Yes | Ta | LG | No |
|  | U39 | 67 | Male |  | Yes | T1 | HG | Yes |
|  | U40 | 69 | Male | 21.50 | Yes | Ta | LG | No |
|  | U41 | 60 | Male |  | Yes | Ta | LG | Yes |
|  | U42 | 72 | Male | 21.09 | Yes | T1 | HG | No |
|  | U43 | 93 | Male | 22.15 | Yes | Ta | LG | Yes |
|  | U44 | 78 | Male | 25.33 | Yes | T1 | HG | Yes |
|  | U46 | 76 | Male | 23.98 | Yes | Ta | HG | Yes |
|  | U47 | 67 | Male |  | Yes | Ta | LG | Yes |
|  | U48 | 84 | Male |  | Yes | Ta | LG | No |
|  | U49 | 72 | Male |  | Yes | NA | LG | No |
|  | U50 | 65 | Male |  | Yes | Ta | LG | No |
|  | U51 | 76 | Male | 23.31 | Yes | Ta | LG | Yes |
|  | U52 | 80 | Male |  | Yes | Ta | LG | No |
|  | U53 | 71 | Male |  | Yes | Ta | LG | Yes |
|  | U54 | 62 | Female | 25.38 | Yes | T1 | HG | Yes |
|  | U55 | 67 | Male | 28.37 | Yes | T1 | HG | No |
|  | U56 | 66 | Male |  | Yes | Ta | HG | Yes |
|  | U57 | 60 | Male | 27.38 | Yes | Ta | LG | Yes |
| **Non CaB** | U24 | 69 | Male | 24.45 |  |  |  |  |
|  | U25 | 76 | Male |  |  |  |  |  |
|  | U26 | 69 | Male | 23.91 |  |  |  |  |
|  | U28 | 63 | Male |  |  |  |  |  |
|  | U29 | 66 | Male | 25.62 |  |  |  |  |
|  | U32 | 71 | Female | 26.44 |  |  |  |  |
|  | U33 | 75 | Male | 24.64 |  |  |  |  |
|  | U34 | 76 | Male | 20.54 |  |  |  |  |
|  | U35 | 74 | Male | 23.42 |  |  |  |  |
|  | U36 | 67 | Female | 26.14 |  |  |  |  |
|  | U37 | 66 | Female |  |  |  |  |  |
|  | U38 | 75 | Male | 22.75 |  |  |  |  |
|  | U58 | 51 | Male |  |  |  |  |  |
|  | U59 | 68 | Male |  |  |  |  |  |
|  | U60 | 64 | Male |  |  |  |  |  |
|  | U61 | 63 | Female | 20.08 |  |  |  |  |
|  | U62 | 63 | Male |  |  |  |  |  |
|  | U63 | 68 | Male | 27.96 |  |  |  |  |
|  | U64 | 52 | Female |  |  |  |  |  |
|  | U65 | 67 | Male |  |  |  |  |  |
